# Supplementary material for: Unraveling surface structures of gallium promoted transition metal catalysts in CO2 hydrogenation
Source: Nat Commun. 2023 Aug 2;14:4649. doi: 10.1038/s41467-023-40361-3 (PMC10397205; doi:10.1038/s41467-023-40361-3)
Supplement: Supplementary file 1 — Supplementary Information [file 41467_2023_40361_MOESM1_ESM.pdf]

Supplementary Information

Unraveling surface structures of gallium promoted transition metal catalysts in CO_2 hydrogenation

Si Woo Lee, Mauricio Lopez Luna, Nikolay Berdunov, Weiming Wan, Sebastian Kunze, Shamil Shaikhutdinov, and Beatriz Roldan Cuenya*

Department of Interface Science, Fritz Haber Institute of the Max Planck Society, 14195 Berlin, Germany

*To whom correspondence should be addressed. E-mail: shaikhutdinov@fhi-berlin.mpg.de

Supplementary Figures

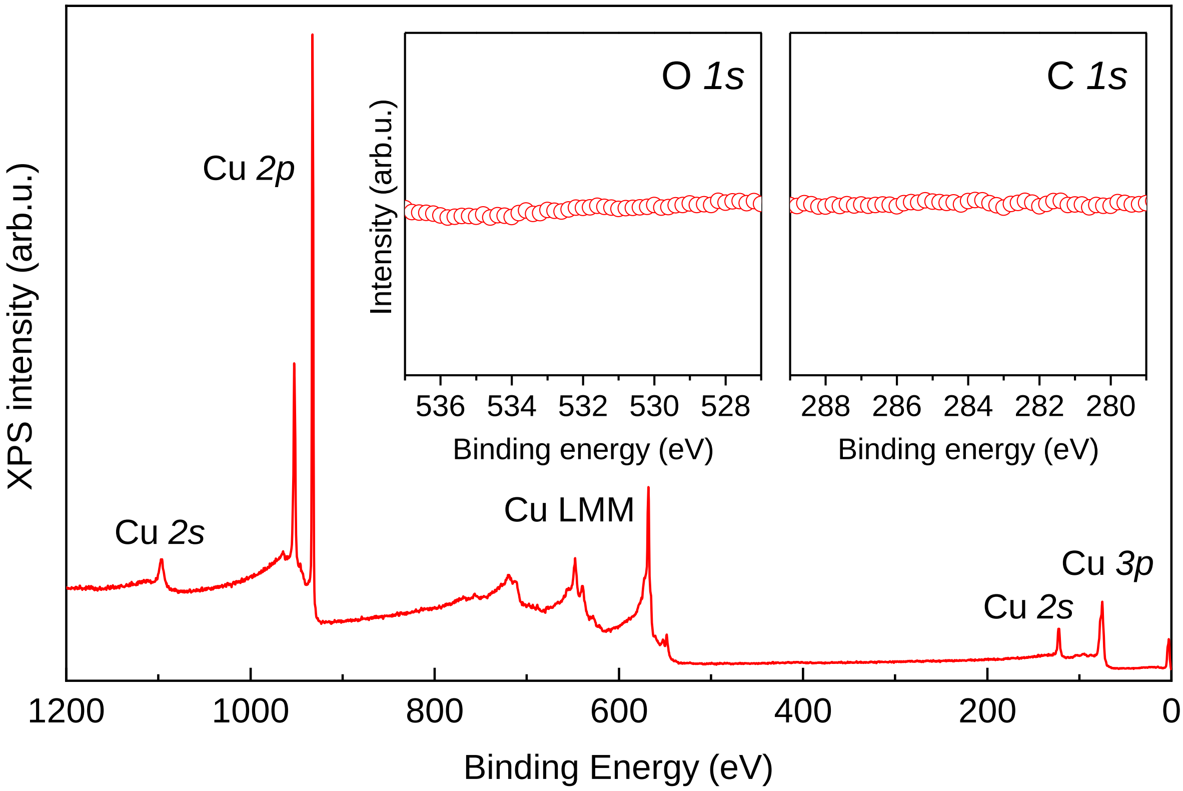

Supplementary Figure 1 | Survey XPS spectrum of the clean Cu(111) surface prepared by several cycles of Ar^+ sputtering and UHV annealing at 900 K. The inset highlights the absence of O and C species.

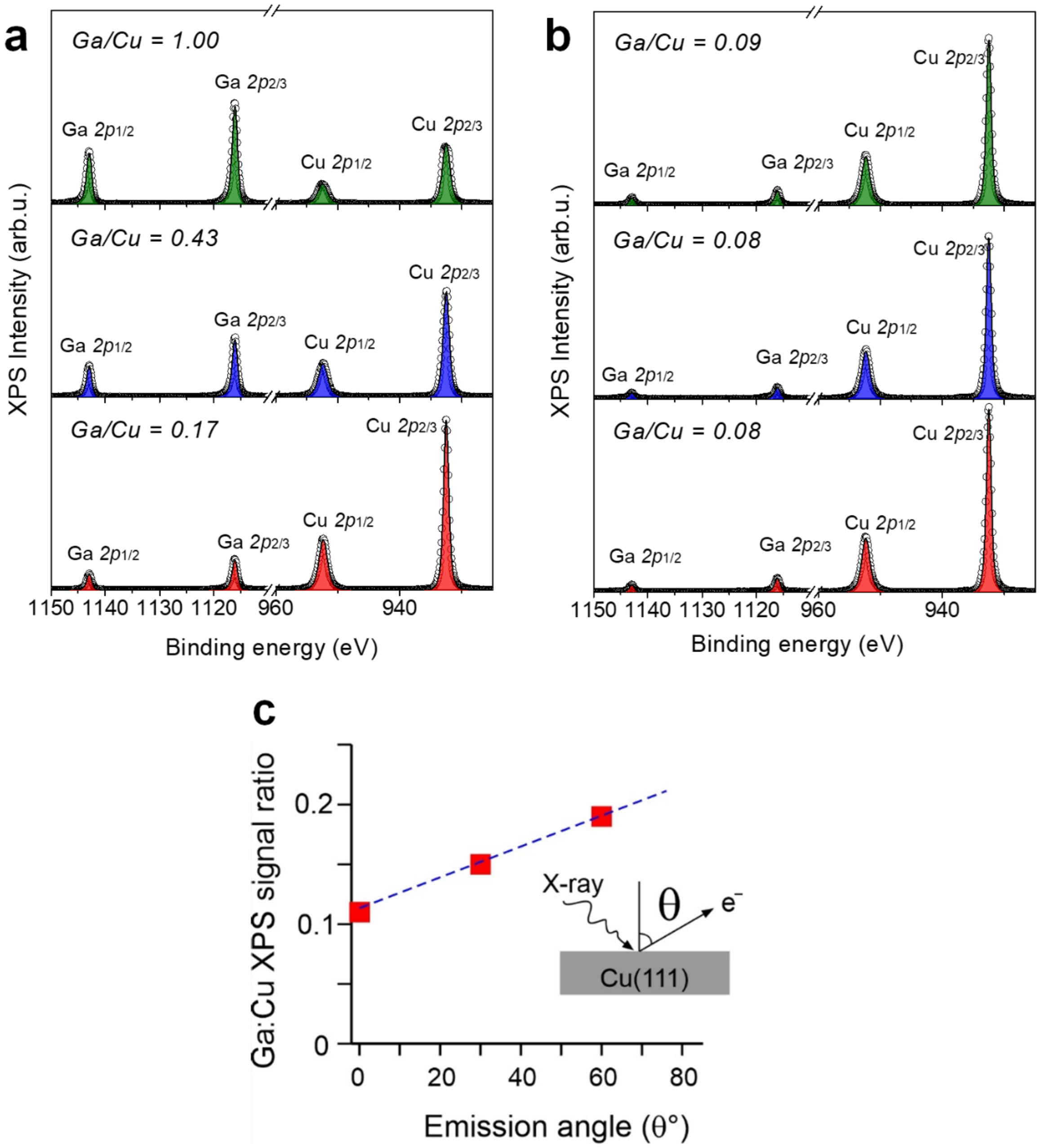

Supplementary Figure 2 | (a,b) Ga *2p* and Cu *2p* regions in the XPS spectra: (**a**) after deposition at 300 K of different amounts of Ga; (**b**) after UHV annealing the same samples at 600 K for 15 min. The calculated Ga:Cu molar ratios using the sensitivity factors provided by CasaXPS are shown adjacent to the spectra. (**c**) Ga *2p* : Cu *2p* signal ratio as a function of the electron emission angle (see inset). The relative increase of the Ga/Cu ratio at grazing emission suggests that Ga is mostly located in the surface layer.

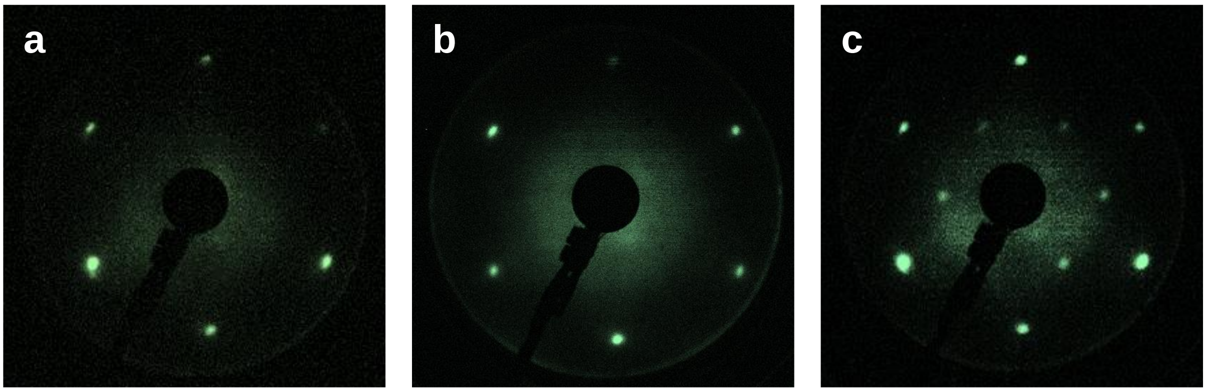

Supplementary Figure 3 | LEED patterns (at 83 eV) of: **a)** the clean Cu(111) surface; **b)** after Ga deposition (Ga/Cu=0.17); **c)** subsequent UHV annealing at 600 K resulting in the Ga($\sqrt{3}\times\sqrt{3}$)R30°-Cu(111) structure.

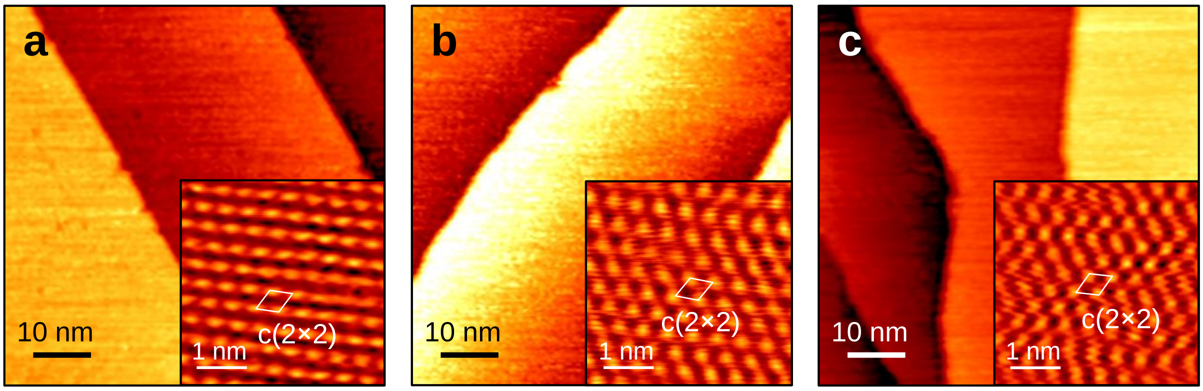

Supplementary Figure 4 | a-c) Room-temperature STM images obtained in UHV after annealing the Ga/Cu(111) samples in UHV at 600 K. The "as deposited" amounts of Ga correspond to the Ga/Cu molar ratios of 0.17 (**a**); 0.43 (**b**), and 1.0 (**c**), see Fig. S2. All samples show the c(2×2) structure in the high-resolution images presented as insets. (Tunneling parameters: sample bias 0.3 V, current 1 nA (**a**); 0.1 V, 7 nA (inset in **a**); 0.5 V, 0.5 nA (**b**); 0.1 V, 8 nA (inset in **b**); and 0.6 V, 0.4 nA (**c**); 0.1 V, 7 nA (inset in **c**).

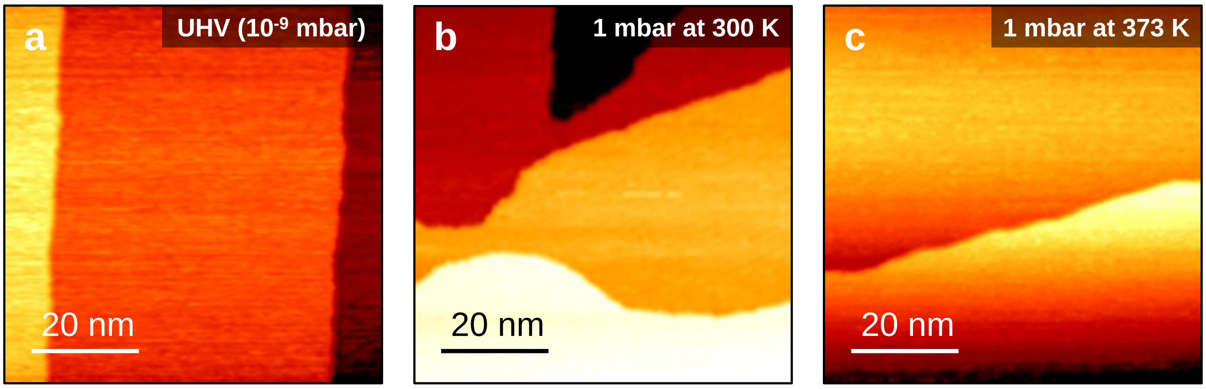

Supplementary Figure 5 | a) STM image obtained in UHV at 300 K on the clean Cu(111) surface. **b-c)** NAP-STM images obtained in the $CO_2 + H_2$ (1:3) reaction mixture at 1 mbar at 300 K (**b**) and 1 mbar at 373 K (**c**). The atomically flat terraces are separated by monoatomic steps of 2 Å in height. Tunneling parameters: 0.4 V, 0.9 nA (**a**); 1V, 0.2 nA (**b**); and 1 V, 0.2 nA (**c**).

In all XPS spectra presented below, every state obtained by deconvolution is shaded in individual color.

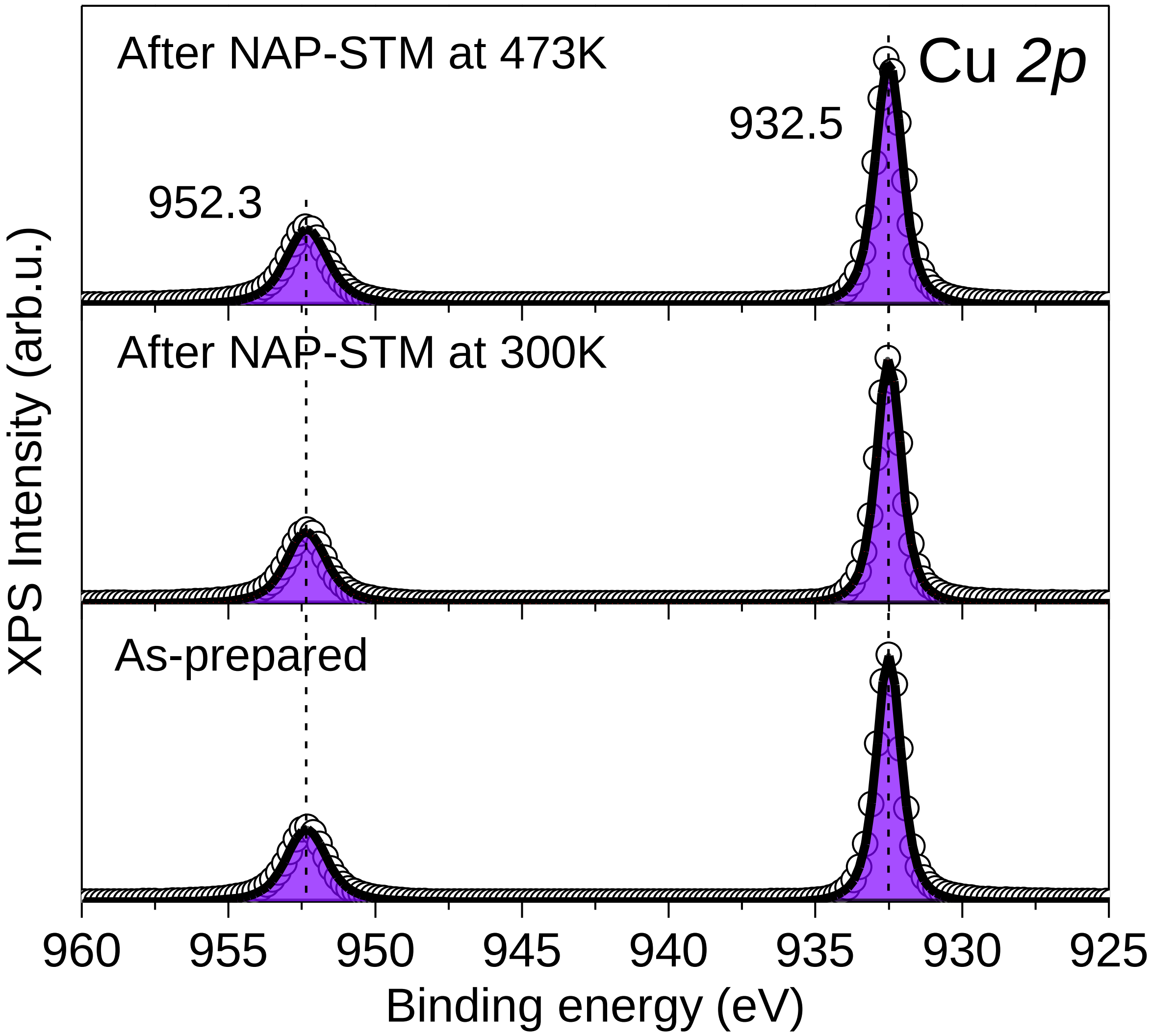

Supplementary Figure 6 | Cu *2p* XPS spectra recorded in UHV before and after the NAP-STM measurements in 1 mbar of the reaction CO_2 + H_2 mixture at 300 K and 473 K as indicated. Cu is metallic in all the samples studied; see also the Cu LMM Auger lines in Fig. 2H of the main text.

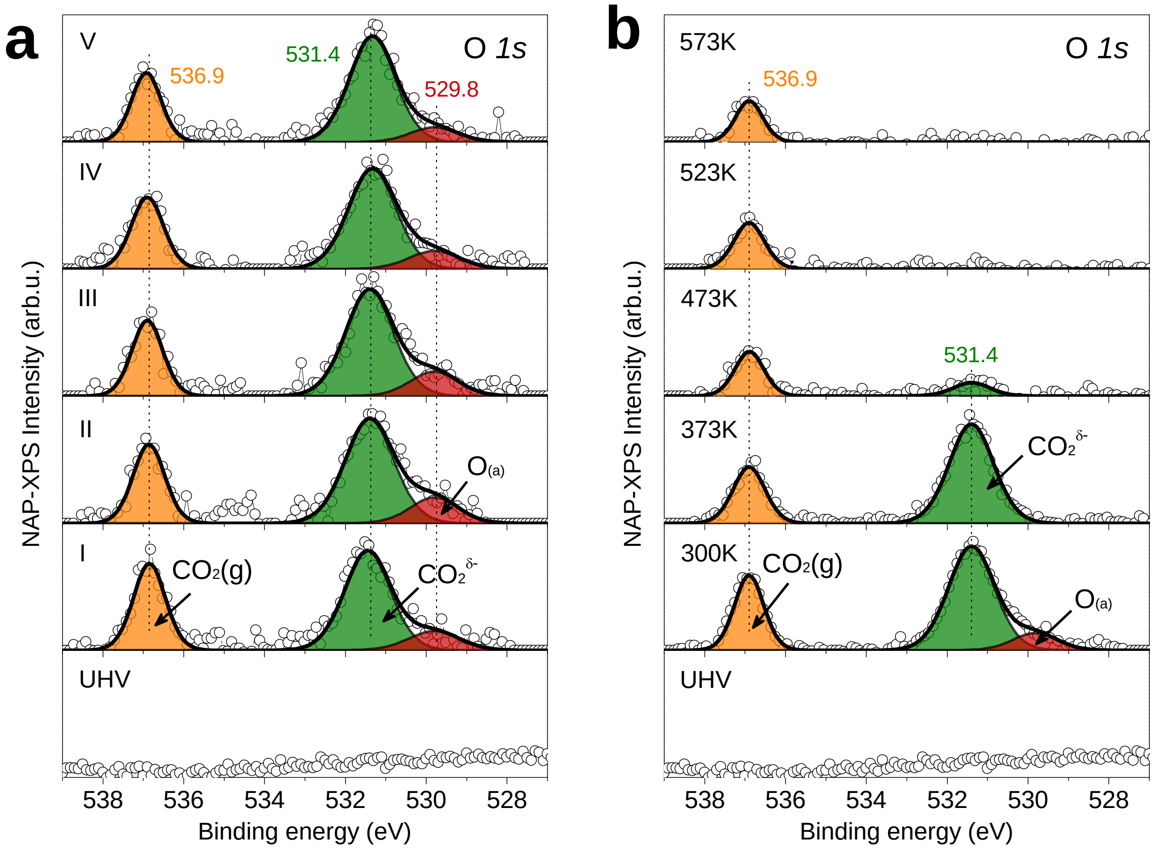

Supplementary Figure 7 | O *1s* NAP-XPS spectra measured in 1 mbar of the reaction CO_2 + H_2 (1:3) mixture on the pure Cu(111) surface: **a)** consecutive spectra (from bottom to top) measured at 300 K; **b)** average of five spectra recorded at different reaction temperatures increased stepwise as indicated. The signal at 536.9 eV originates from CO_2 in the gas phase. The 531.4 eV signal is assigned to CO_2 adsorption via carboxylate ($CO_2^{\delta-}$), and the 529.8 eV signal to the O ad-atoms.

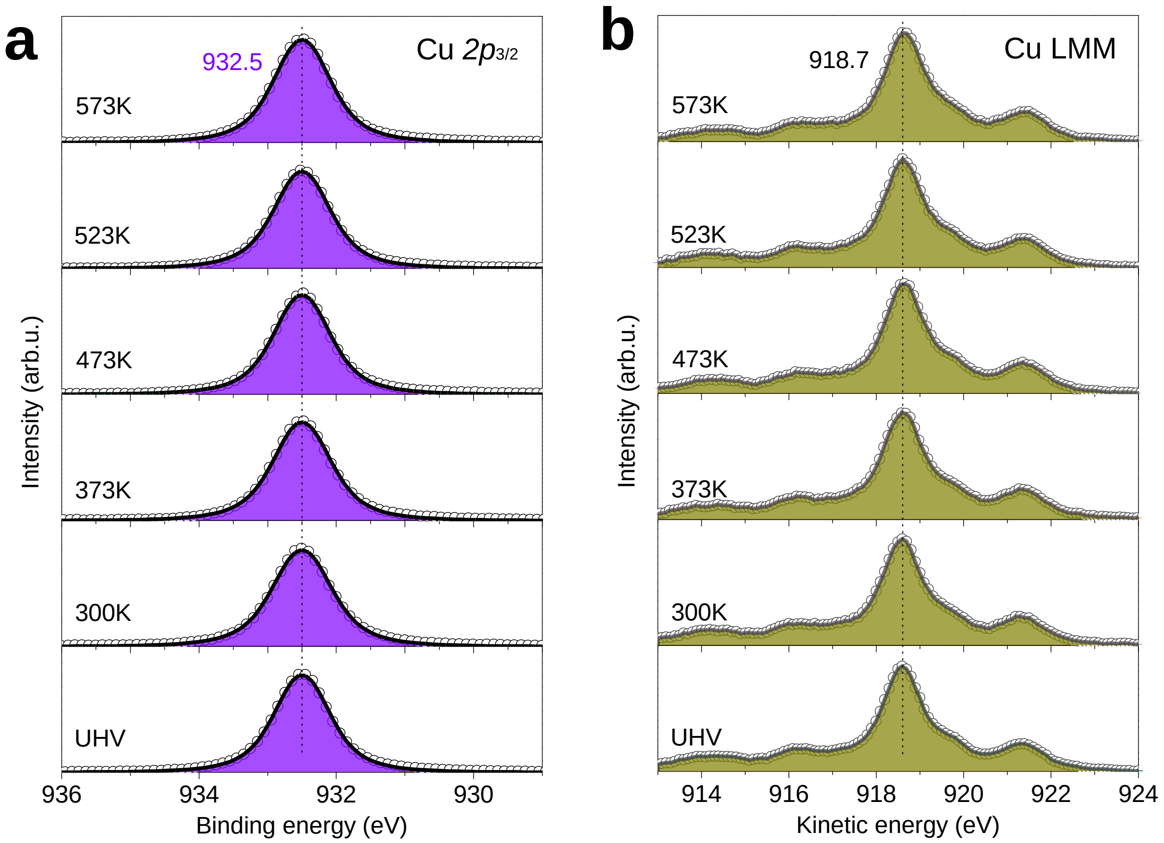

Supplementary Figure 8 | a) Cu *2p*$_{3/2}$ spectra and **b)** Cu LMM Auger lines complementary to the Ga *2p* and O *1s* spectra shown in Fig. 3 of the main text, measured in UHV (bottom line) and in 1 mbar of the reaction CO_2 + H_2 (1:3) mixture at different temperatures increased stepwise as indicated. The results show that Cu remains metallic under the reaction conditions studied.

Supplementary Figures 9-14 below illustrate that basically the same spectral evolution is observed for all c(2×2)Ga-Ga(111) samples studied, irrespectively of the initial Ga coverage. The difference is only the time and temperature, at which Ga became fully oxidized. In particular, the "high coverage" sample was more resistant towards oxidation, which could only be completed at 373 K (Supplementary Figs. 12,13). Such behavior can be rationalized within the scenario described in the main text, where Ga passivates the Cu surface, and the oxidation initially occurs via the reaction with the O atoms produced on the Ga-free, clean Cu regions, whose presence is obviously minimized on samples with initially high Ga coverage.

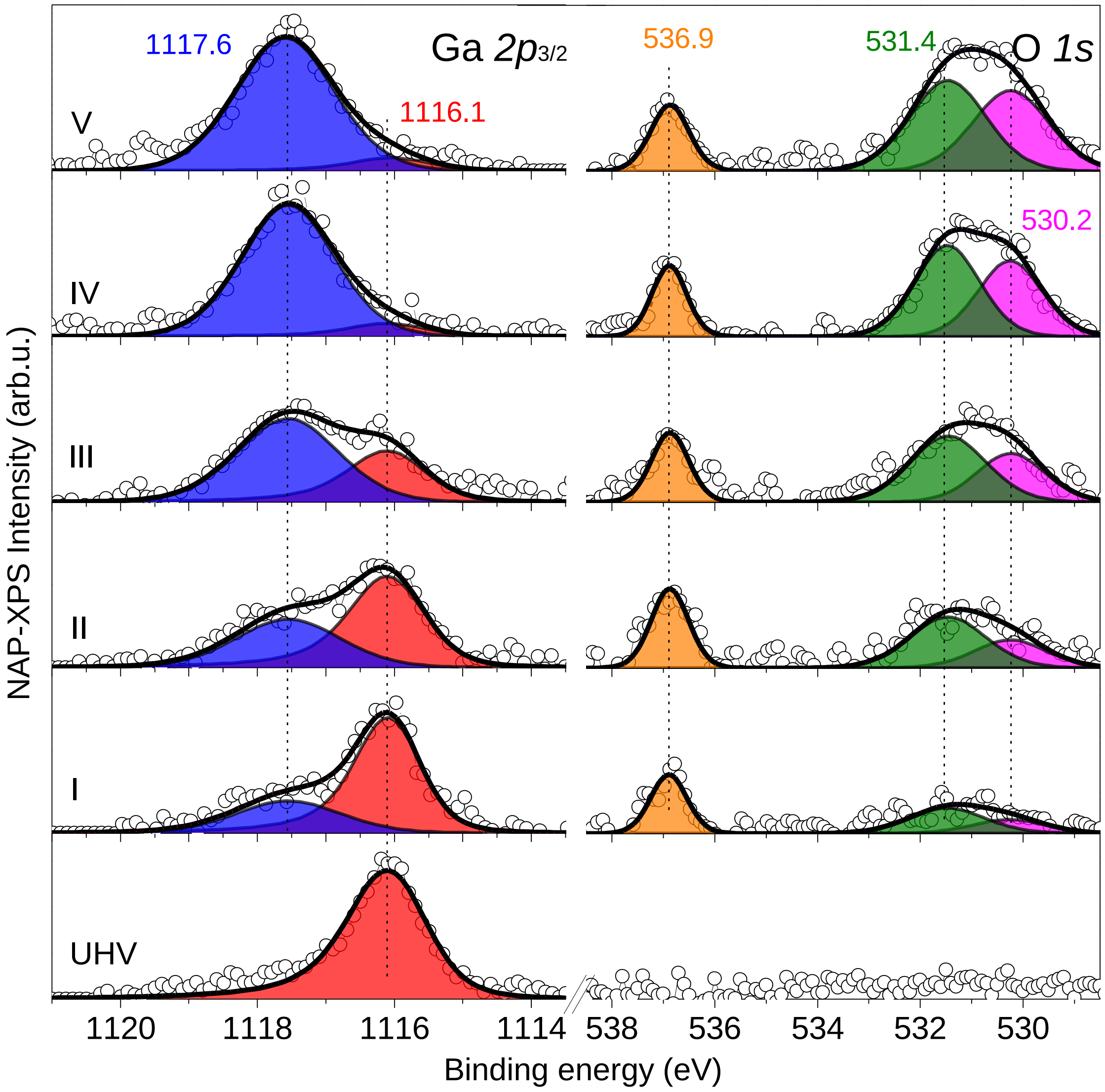

Supplementary Figure 9 | From bottom to top: Consecutive Ga $2p_{3/2}$ and O *1s* NAP-XPS spectra obtained on the c(2×2)-Ga/Cu(111) surface (the Ga/Cu ratio in the "as deposited" sample was 0.43) in 1 mbar of $CO_2 + H_2$ (1:3) at 300 K.

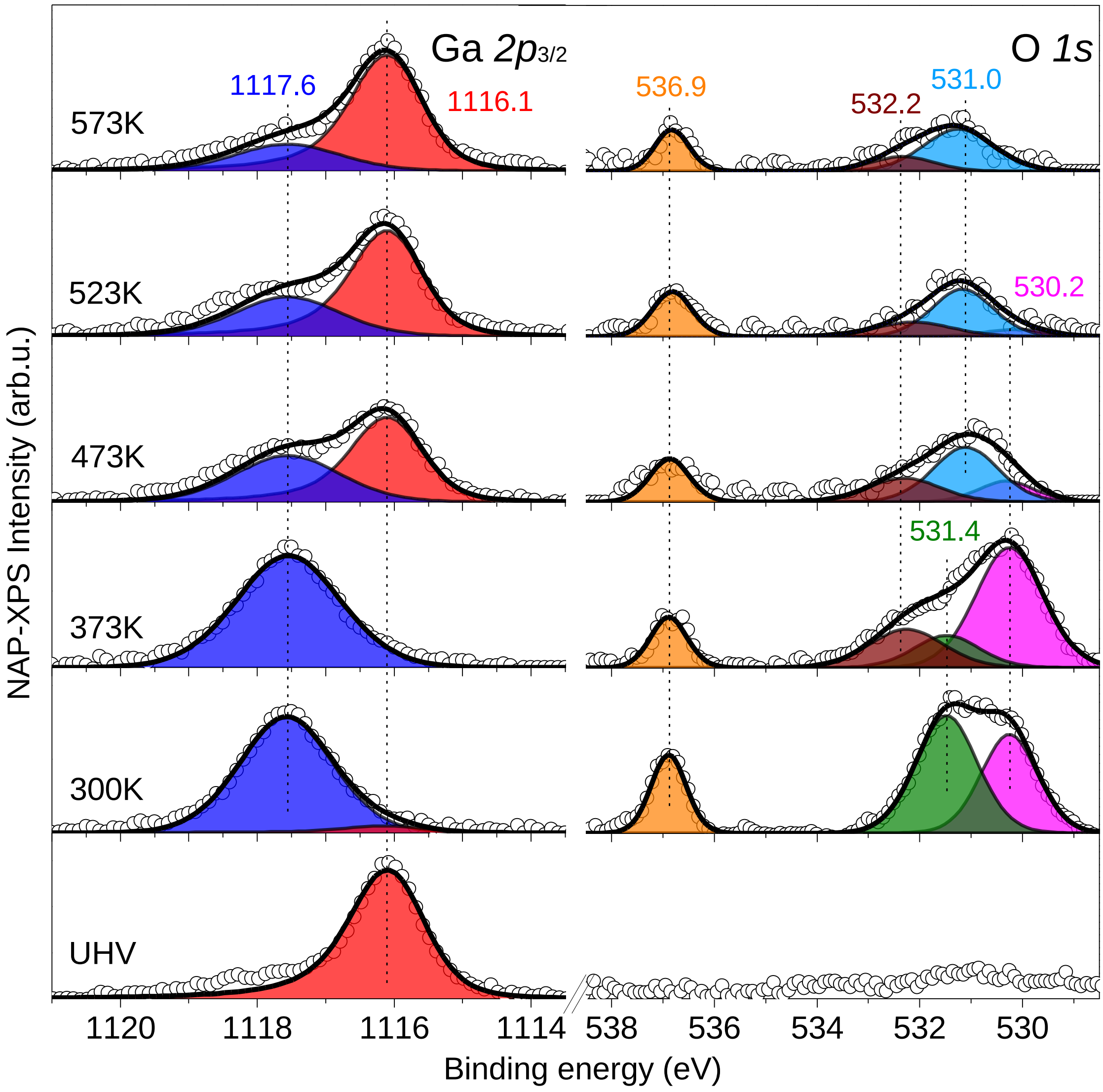

Supplementary Figure 10 | Ga *2p*$_{3/2}$ and O *1s* regions in NAP-XPS spectra obtained on the c(2×2)-Ga/Cu(111) surface (the Ga/Cu ratio in the "as deposited" sample was 0.43) in 1 mbar of CO_2 + H_2 (1:3) at reaction temperatures increased stepwise as indicated.

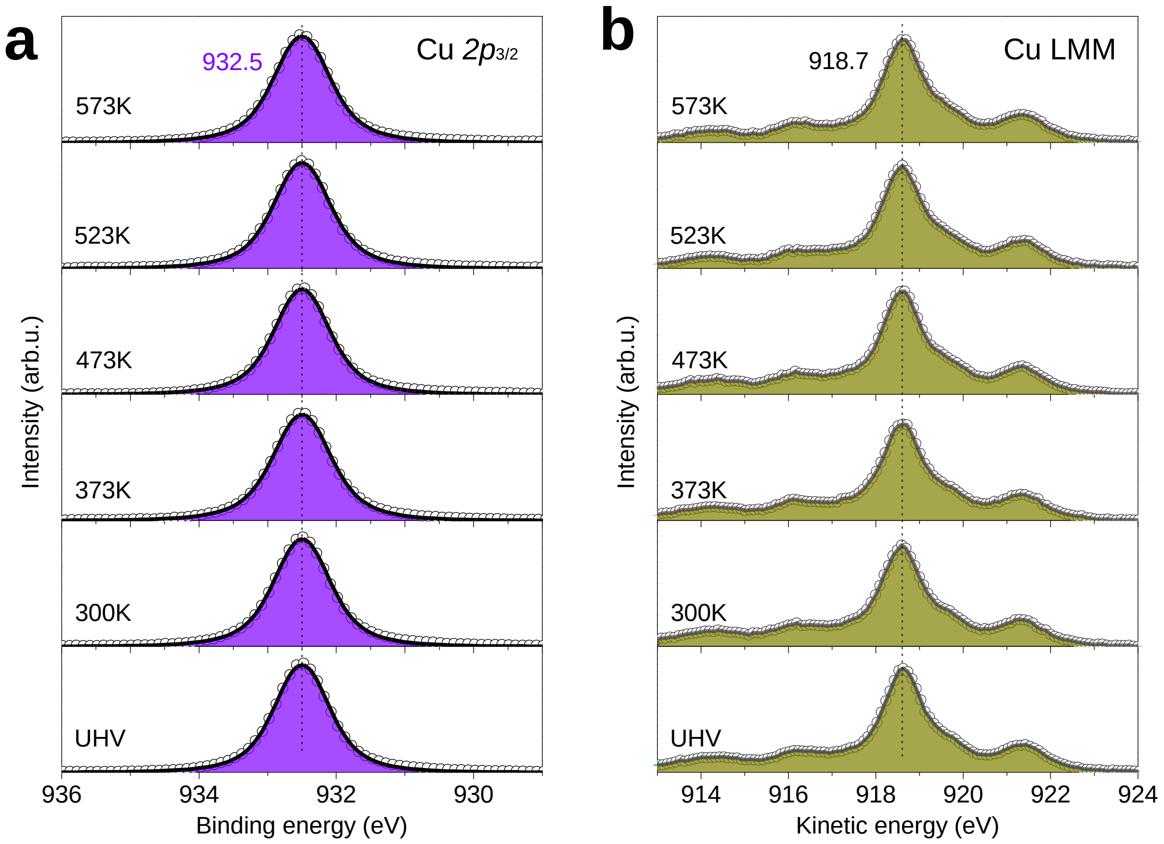

Supplementary Figure 11 | a) Cu *2p*$_{3/2}$ and **b)** Cu LMM Auger lines in the NAP-XPS spectra obtained on the c(2×2)-Ga/Cu(111) surface (the Ga/Cu ratio in the "as deposited" sample was 0.43) in 1 mbar of $CO_2 + H_2$ (1:3) at reaction temperature increased stepwise as indicated.

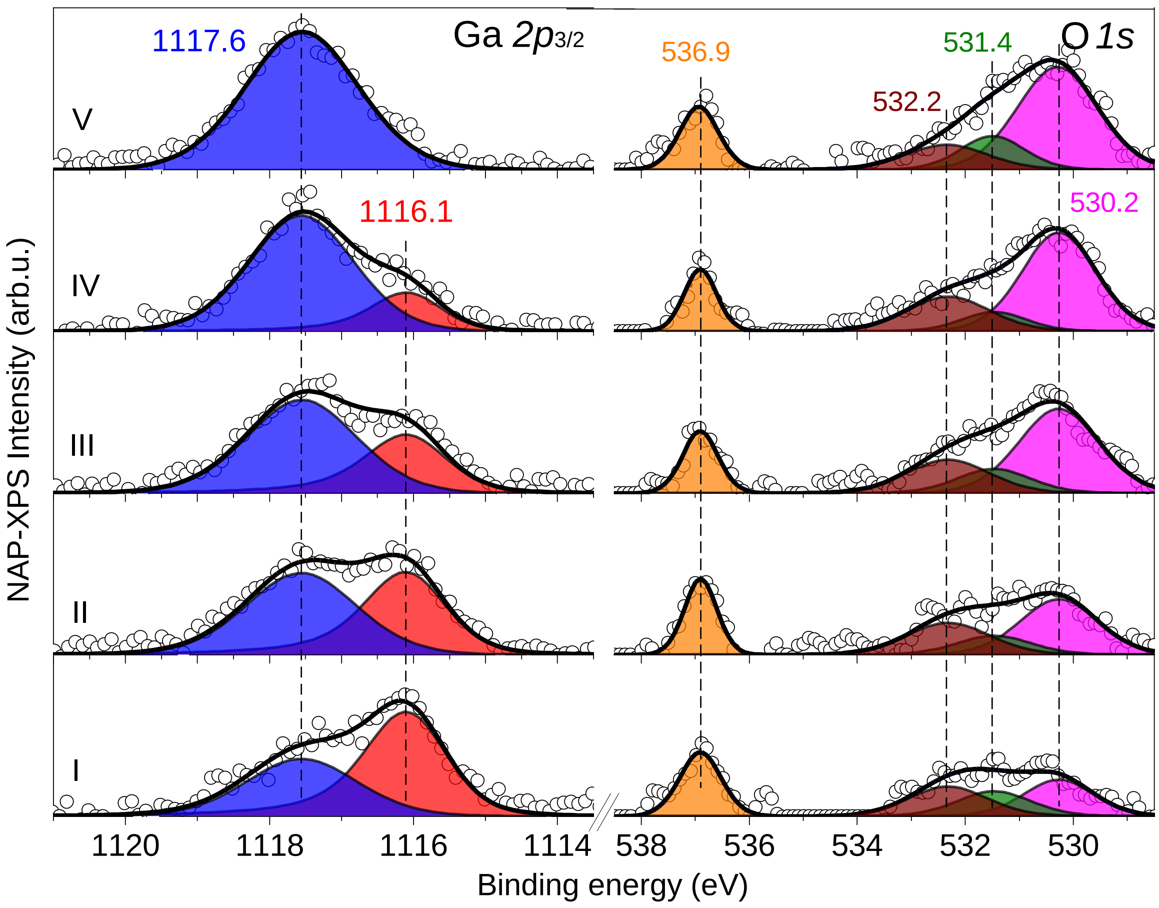

Supplementary Figure 12 | From bottom to top: Consecutive Ga $2p_{3/2}$ and O *1s* NAP-XPS spectra obtained on the c(2×2)-Ga/Cu(111) surface (the Ga/Cu ratio in the “as deposited” sample was 1.0) in 1 mbar of $CO_2 + H_2$ (1:3) at 373 K.

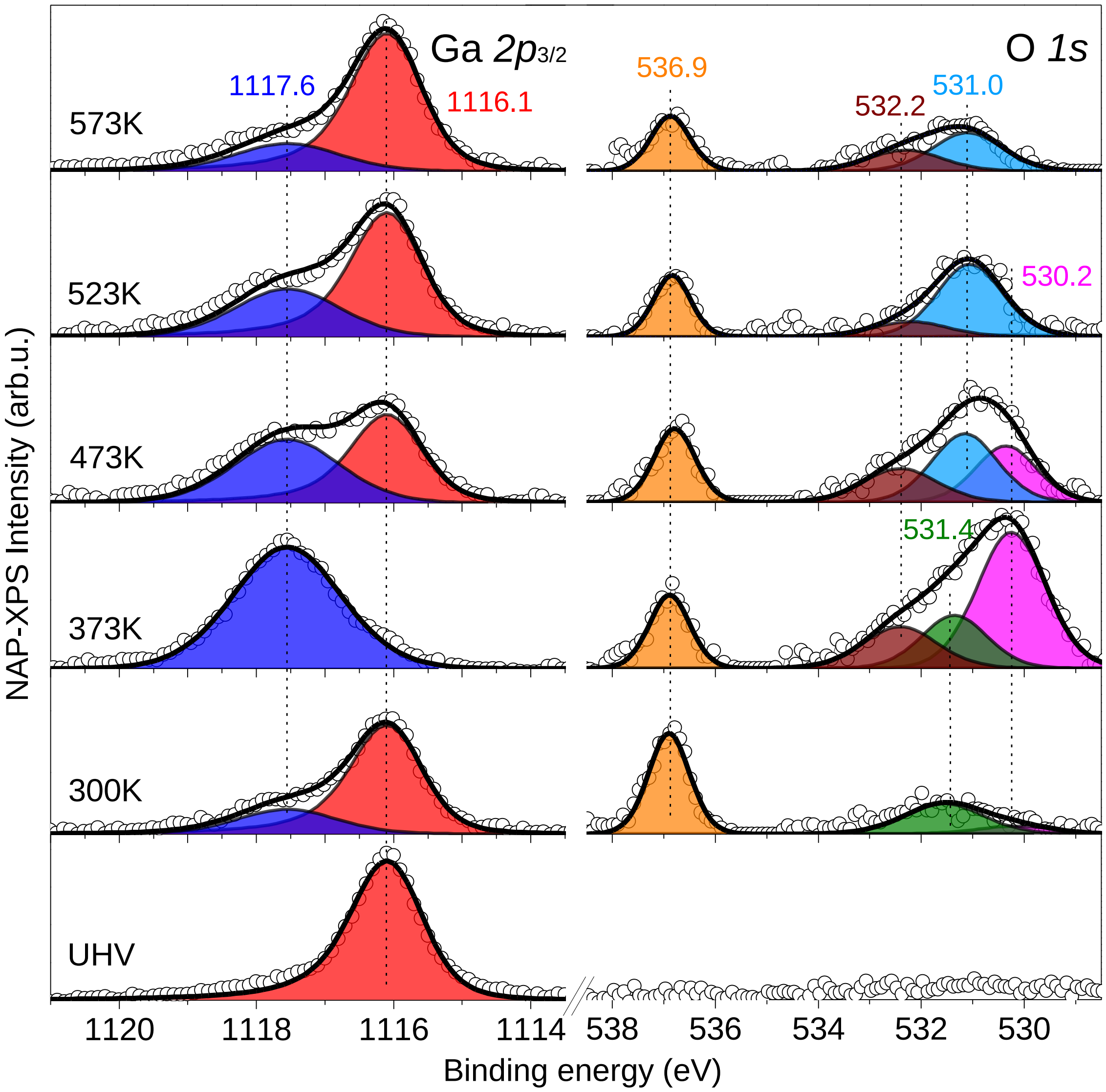

Supplementary Figure 13 | Ga *2p*$_{3/2}$ and O *1s* regions in NAP-XPS spectra obtained on the c(2×2)-Ga/Cu(111) surface (the Ga/Cu ratio in the "as deposited" sample was 1.0) in 1 mbar of $CO_2 + H_2$ (1:3) at reaction temperatures increased stepwise as indicated.

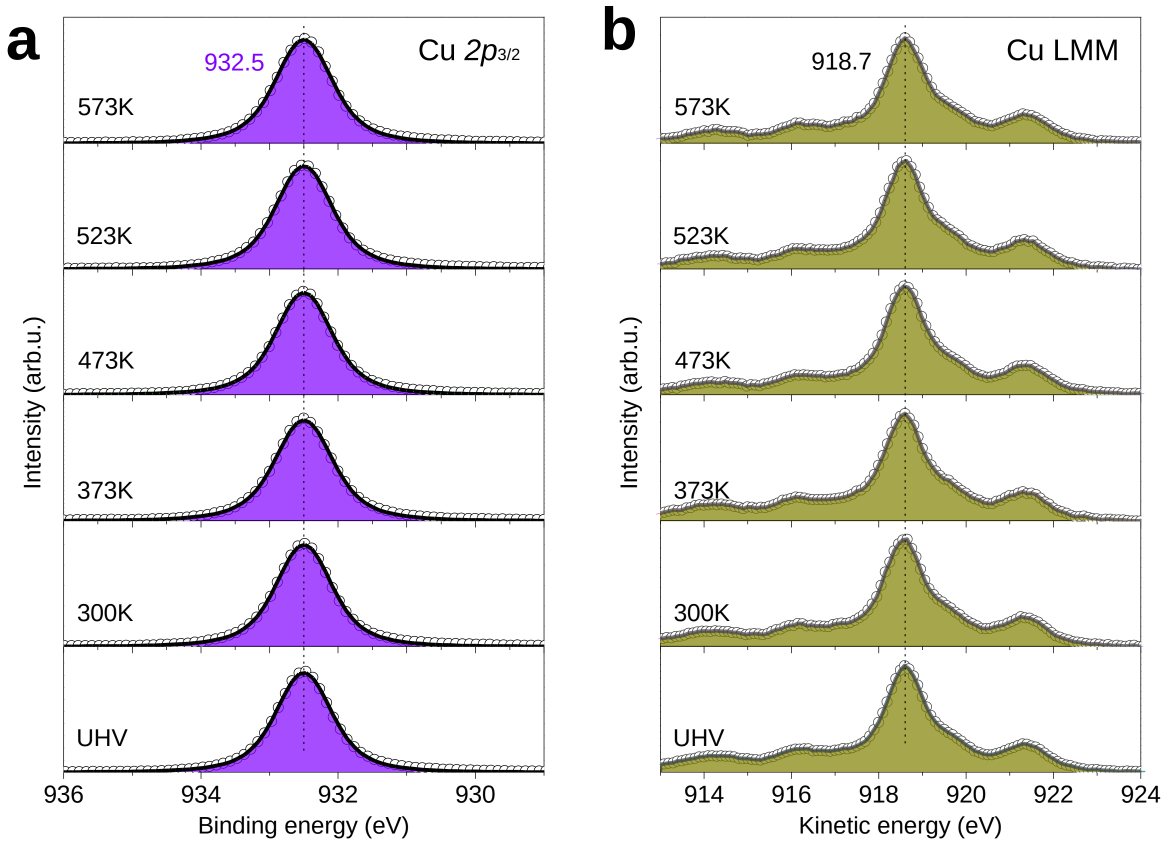

Supplementary Figure 14 | a) Cu *2p*$_{3/2}$ and **b)** Cu LMM Auger lines in the NAP-XPS spectra obtained on the c(2×2)-Ga/Cu(111) surface (the Ga/Cu ratio in the "as deposited" sample was 1.0) in 1 mbar of CO_2 + H_2 (1:3) at reaction temperature increased stepwise as indicated.

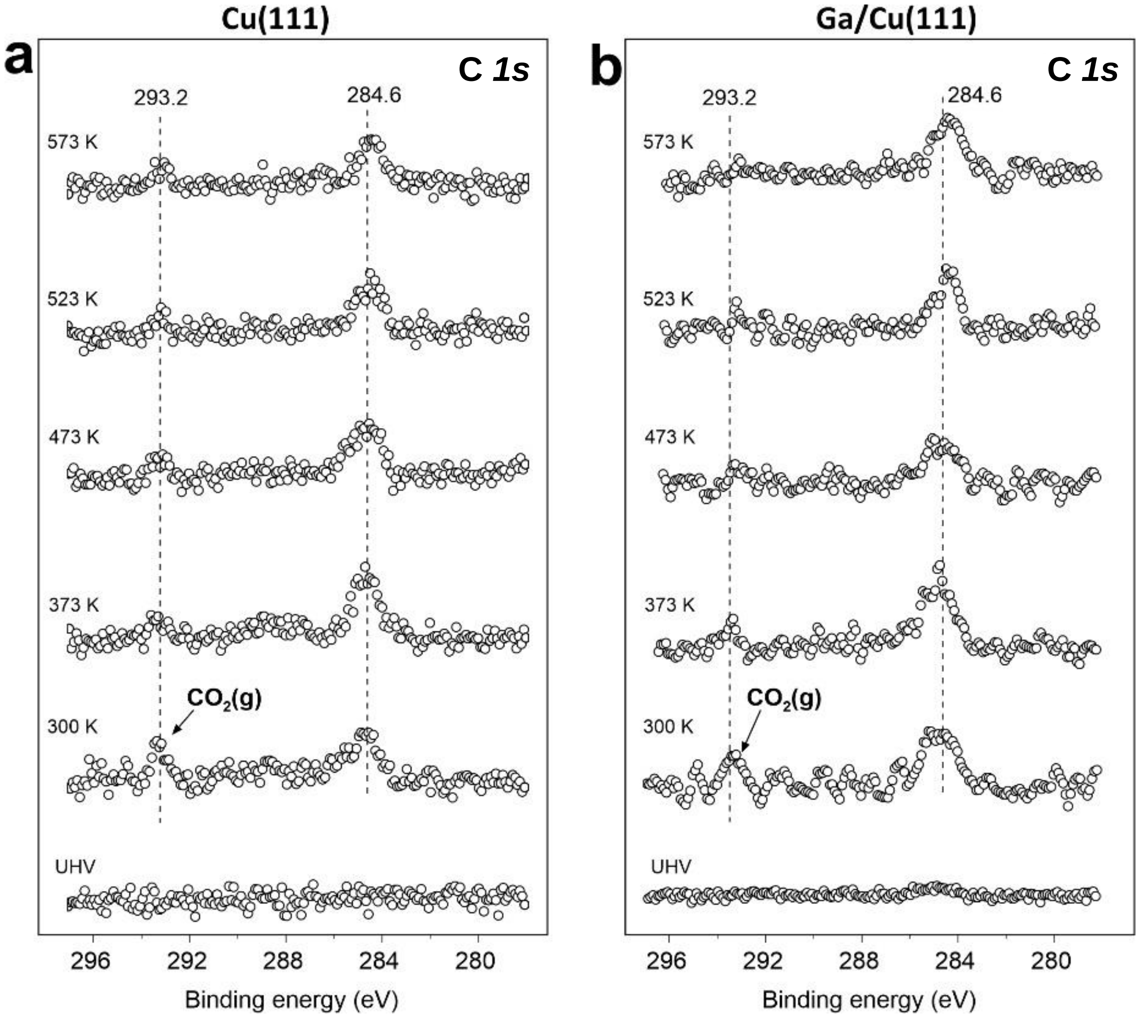

Supplementary Figure 15 | The C *1s* region in the NAP-XPS spectra measured in 1 mbar of the CO_2 + H_2 (1:3) reaction mixture on pure Cu(111) (**a**) and Ga-Cu(111) (**b**) surfaces at different temperatures increased stepwise as indicated (from bottom to top). The corresponding O *1s* spectra are displayed in Supplementary Figure 7 and Figure 3b in the main text, for Cu and Ga-Cu surfaces, respectively. The signal at 293.2 eV originates from CO_2 in the gas phase. The peak at 284.6 eV is assigned to adventitious carbon.[1]

Supplementary Note 1: In the experiments described below in relation to Supplementary Fig. 16, two identically prepared Ga-Cu samples were respectively exposed to a $CO_2 + H_2$ mixture and pure CO_2 under the same conditions (1 mbar, 473 K, 30 min) and then measured by XPS in UHV at 300 K. The spectra showed that Ga is fully oxidized in both samples, while Cu becomes oxidized in pure CO_2 forming thin oxide layer that does not adsorb CO_2 (see also refs. [2,3]), but remains metallic in $CO_2 + H_2$ (Supplementary Fig. 15c). The corresponding O *1s* spectrum (Supplementary Fig. 15b) of the Ga-Cu sample treated in pure CO_2 showed a very weak signal at around 532 eV assigned to carbonate species. However, this signal is an order of magnitude smaller than that of the same sample treated in $CO_2 + H_2$. Therefore, the prominent 532.2 eV signal observed in $CO_2 + H_2$ can hardly be assigned to carbonates.

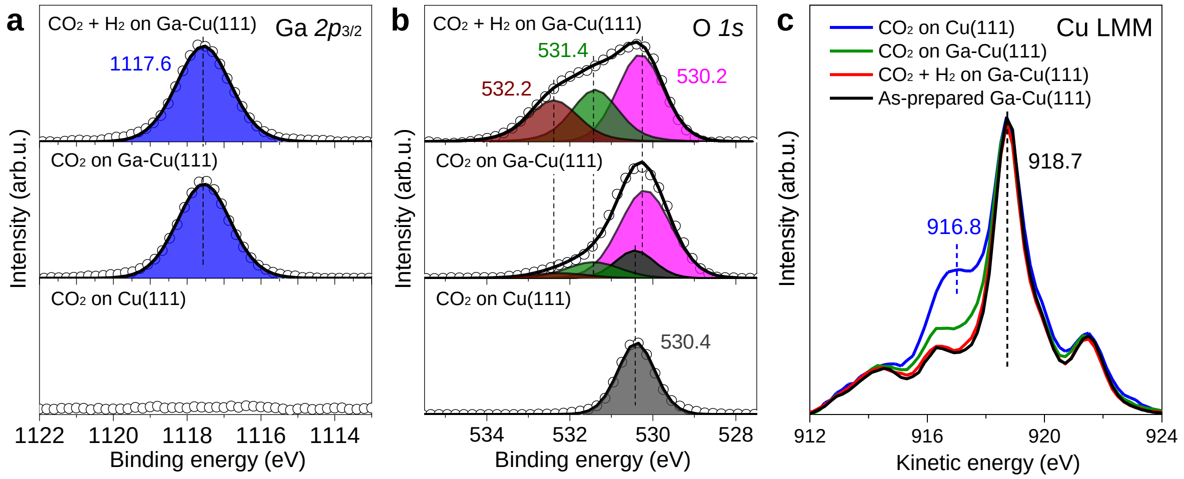

Supplementary Figure 16 | a) Ga $2p_{3/2}$, **b)** O *1s* spectra, and **c)** Cu LMM Auger lines measured in UHV at 300 K on the identically prepared Ga-Cu(111) samples after exposure to $CO_2 + H_2$ mixture and pure CO_2 (total 1 mbar, 473 K, 30 min) as indicated. For comparison, the results on Cu(111) in pure CO_2 are also shown. The Auger spectra were normalized to the maximum at 918.7 eV to better see the prominent signal centered at 916.8 eV associated with Cu^+ in the "monolayer" CuO_x surface oxide formed in pure CO_2, also reflected by the O *1s* peak at 530.4 eV. The CuO_x oxide film reduces back to Cu metal in the $CO_2 + H_2$ ambient. The O *1s* signal at 531.4 eV is assigned to carboxylate ($CO_2^{\delta-}$) on metallic Cu(111).

Supplementary Note 2: In the experiments related to Suppl. Fig. 17, the Ga-Cu sample treated in pure CO_2 was flashed to 500 K in UHV to desorb CO_2-related adsorbates and subsequently exposed to 1 mbar of H_2 at 473 K for 30 min to hydroxylate the Ga oxide surface formed by CO_2 adsorption. A prominent signal at 531.8 eV, i.e., shifted by 1.6 eV with respect to oxygen in oxide (530.2 eV), falls in the range of the BE shifts (1.5 – 2.0 eV) reported for hydroxylated surfaces of the transition metal oxides[4] and thus assigned to hydroxyls. Therefore, the 532.2 eV signal observed in CO_2 + H_2 can hardly be assigned to hydroxyls. In fact, this state bears close similarity to that observed on the Cu surfaces modified with ZnO and CeO_2 and assigned to formate[5-7]. Hydroxyls cannot be the origin of the 531.0 eV state appearing in the NAP experiments at high temperatures. It is tempting to assign the 531.0 eV signal to methoxy species, as it falls in the range observed for methoxy formed by methanol adsorption[8,9]. On the other hand, this state appears along with the Ga-oxide partial reduction (Fig. 3b) and may therefore be related to the O-deficient GaO_x domains.

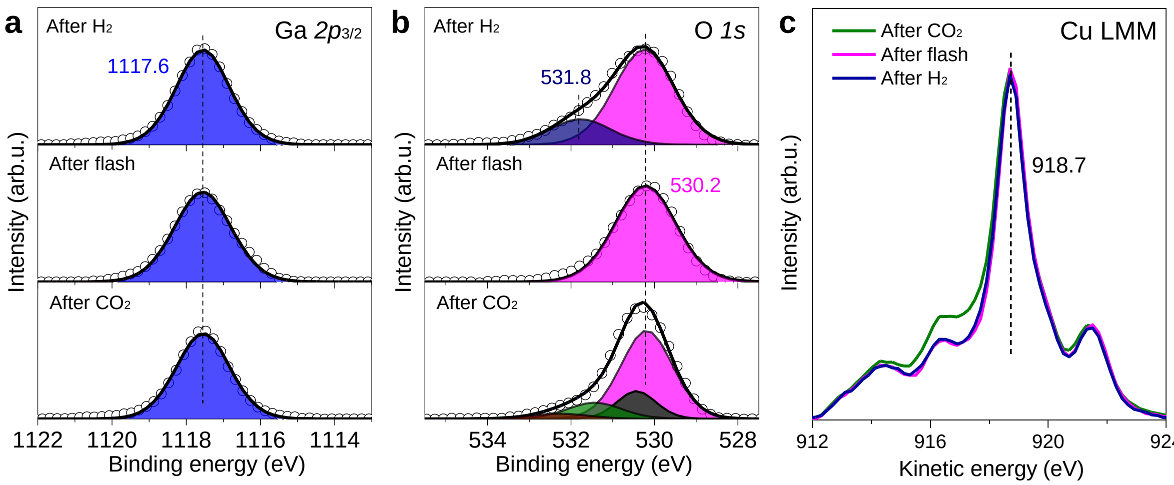

Supplementary Figure 17 | a) Ga *2p*$_{3/2}$, **b)** O *1s* spectra, and **c)** Cu LMM Auger lines measured in UHV at 300 K on the Ga-Cu(111) sample after exposure to pure CO_2 (total 1 mbar, 473 K, 30 min), then flashed to 500 K in UHV, and after subsequent exposure to H_2 (1 mbar, 473 K, 30 min). The Auger spectra were normalized to the maximum at 918.7 eV. The signal at 916.8 eV associated with Cu^+ in the "monolayer" CuO_x surface oxide formed in pure CO_2 disappears due to reduction to Cu metal. The O *1s* peak at 531.8 eV is assigned to surface hydroxyls on the Ga-oxide surface.

Supplementary Note 3: To evaluate whether the Ga-Cu surface undergoes the same structural/chemical evolution when exposed to more catalytically relevant pressures, our model catalysts were exposed to the CO_2 + H_2 (1:3) reaction mixture at 5 bar (total) in a high-pressure cell (see Methods) at the same temperatures and exposure times as in the NAP-XPS experiments. Then, the sample was cooled down to room temperature, the reactor cell was pumped out, and the sample was transferred to the XPS/STM chamber without exposure to air.

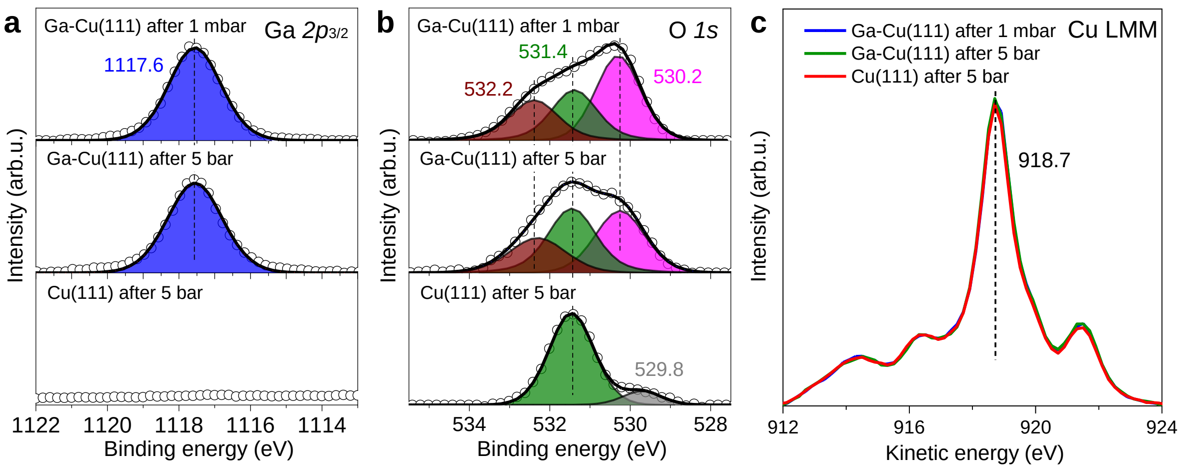

Supplementary Figure 18 | a) Ga *2p*$_{3/2}$, **b)** O *1s*, and **c)** Auger Cu LMM regions in XPS spectra recorded in UHV at 300 K on Cu(111) and Ga/Cu(111) samples after exposure to CO_2 + H_2 (1:3) atmosphere at 5 bar (total) at temperatures increased stepwise from 300 K to 573 K in the same manner as used in NAP-XPS measurements shown in Fig. 3. The spectra recorded in UHV at 300 K on the Ga/Cu(111) sample after reaction at 1 mbar are shown for comparison. The O *1s* peak at 531.4 eV is assigned to carboxylate ($CO_2^{\delta-}$), and the weak signal at 529.8 eV - to O ad-atoms on the Cu(111) surface. The 530.2 and 532.2 eV signals (only observed on the Ga-Cu surfaces) are assigned, respectively, to lattice oxygen (O^{2-}) in GaO_x and formate ($HCOO^-$) on GaO_x. The Cu(111) surface remains metallic in all these experiments as evidenced by the Auger Cu LMM lines (see also Supplementary Figs. 15-16)

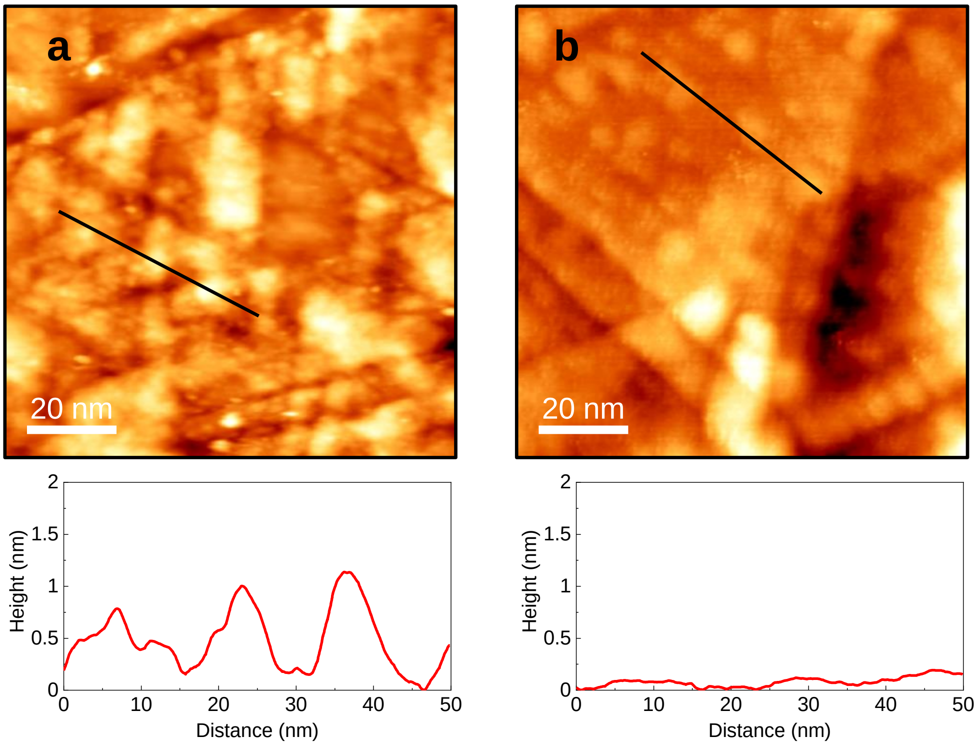

Supplementary Figure 19 | STM images of the Ga-Cu(111) (**a**) and Cu(111) (**b**) samples after experiments at 5 bar used in Supplementary Fig. 17. The topography profiles along the black lines are shown below the images. Apparently, the reacted Ga-Cu surface exhibits a much higher corrugation amplitude as compared to that for pure Cu.

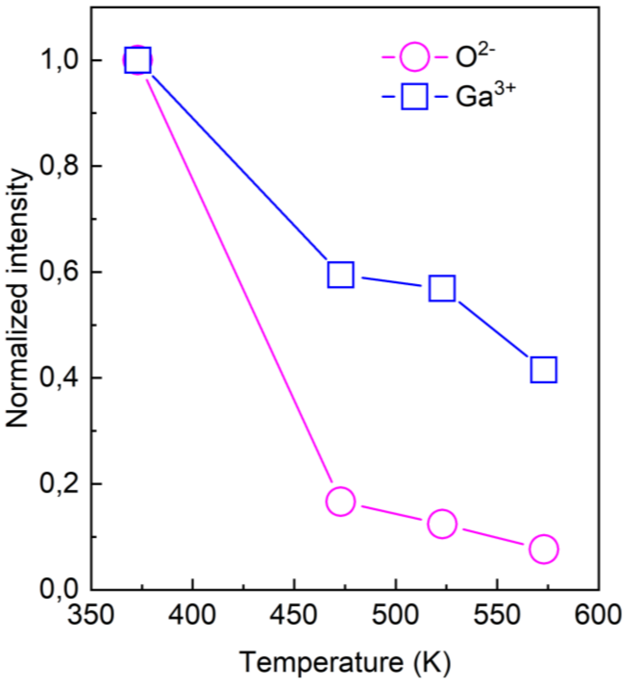

Supplementary Figure 20 | The fraction of Ga^{3+} in total amounts of Ga at the surface and intensity of the lattice oxygen (O^{2-}) signal at 530.2 eV, determined from the spectra shown in Fig. 3 of the main text and normalized to the maximum values, as a function of the sample temperature in 1 mbar of CO_2 + H_2 (1:3) reaction mixture. It is clear that the signal from the lattice oxygen decreases to a considerably larger extent than the degree of reduction measured from the Ga *2p* spectra. This finding indicates that a large fraction of the O atoms in GaO_x is involved in the chemisorption of the ambient gases and hence, is located at the surface. As a result, the corresponding O^{2-} signal attenuates at the expense of the adsorbate-related signals.

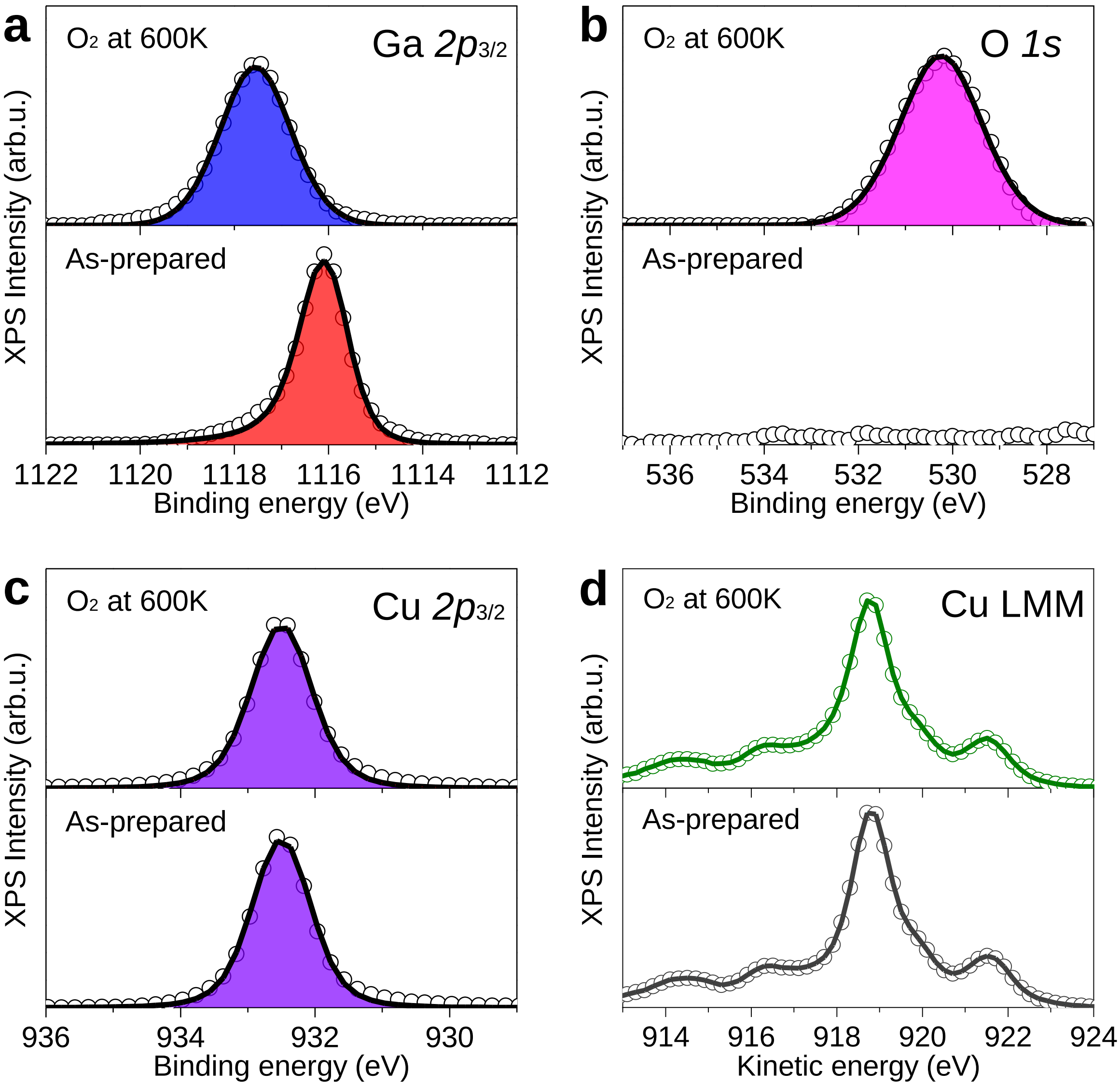

Supplementary Figure 21 | a) Ga $2p_{3/2}$, **b)** O $1s$, **c)** Cu $2p_{3/2}$, and **d)** Cu LMM Auger lines in the XPS spectra measured in UHV on the c(2×2)Ga-Cu(111) before and after annealing in 10^{-6} mbar of O_2 at 600 K for 15 min.

Supplementary References

1 Deng, X. *et al.* Surface chemistry of Cu in the presence of CO_2 and H_2O. *Langmuir* **24**, 9474-9478 (2008).

2 Eren, B., Weatherup, R. S., Liakakos, N., Somorjai, G. A. & Salmeron, M. Dissociative carbon dioxide adsorption and morphological changes on Cu(100) and Cu(111) at ambient pressures. *J. Am. Chem. Soc.* **138**, 8207-8211 (2016).

3 Yang, T. *et al.* Surface orientation and pressure dependence of CO_2 activation on Cu surfaces. *J. Phys. Chem. C* **124**, 27511-27518 (2020).

4 Dupin, J.-C., Gonbeau, D., Vinatier, P. & Levasseur, A. Systematic XPS studies of metal oxides, hydroxides and peroxides. *Phys. Chem. Chem. Phys.* **2**, 1319-1324 (2000).

5 Graciani, J. *et al.* Highly active copper-ceria and copper-ceria-titania catalysts for methanol synthesis from CO_2. *Science* **345**, 546-550 (2014).

6 Senanayake, S. D. *et al.* Hydrogenation of CO_2 to methanol on CeO_x/Cu (111) and ZnO/Cu (111) catalysts: role of the metal–oxide interface and importance of Ce^{3+} sites. *J. Phys. Chem. C* **120**, 1778-1784 (2016).

7 Palomino, R. M. *et al.* Hydrogenation of CO_2 on ZnO/Cu (100) and ZnO/Cu (111) catalysts: role of copper structure and metal–oxide interface in methanol synthesis. *J. Phys. Chem. B* **122**, 794-800 (2018).

8 Goodacre, D. *et al.* Methanol Adsorption on Vanadium Oxide Surfaces Observed by Ambient Pressure X-ray Photoelectron Spectroscopy. *J. Phys. Chem. C* **125**, 23192-23204 (2021).

9 Orozco, I. *et al.* *In situ* studies of methanol decomposition over Cu (111) and Cu_2O/Cu (111): Effects of reactant pressure, surface morphology, and hot spots of active sites. *J. Phys. Chem. C* **125**, 558-571 (2020).
